# ChatGPT's Epoch in Rheumatological Diagnostics: A Critical Assessment in the Context of Sjögren's Syndrome

**DOI:** 10.7759/cureus.47754

**Published:** 2023-10-26

**Authors:** Bilal Irfan, Aneela Yaqoob

**Affiliations:** 1 Microbiology and Immunology, University of Michigan, Ann Arbor, USA; 2 Infectious Diseases, Beaumont Hospital, Taylor, USA

**Keywords:** artificial intelligence (ai), large language models (llms), chatgpt-4, chatgpt, sjögren’s syndrome

## Abstract

Introduction: The rise of artificial intelligence in medical practice is reshaping clinical care. Large language models (LLMs) like ChatGPT have the potential to assist in rheumatology by personalizing scientific information retrieval, particularly in the context of Sjögren's Syndrome. This study aimed to evaluate the efficacy of ChatGPT in providing insights into Sjögren's Syndrome, differentiating it from other rheumatological conditions.

Materials and methods: A database of peer-reviewed articles and clinical guidelines focused on Sjögren's Syndrome was compiled. Clinically relevant questions were presented to ChatGPT, with responses assessed for accuracy, relevance, and comprehensiveness. Techniques such as blinding, random control queries, and temporal analysis ensured unbiased evaluation. ChatGPT's responses were also assessed using the 15-questionnaire DISCERN tool.

Results: ChatGPT effectively highlighted key immunopathological and histopathological characteristics of Sjögren's Syndrome, though some crucial data and citation inconsistencies were noted. For a given clinical vignette, ChatGPT correctly identified potential etiological considerations with Sjögren's Syndrome being prominent.

Discussion: LLMs like ChatGPT offer rapid access to vast amounts of data, beneficial for both patients and providers. While it democratizes information, limitations like potential oversimplification and reference inaccuracies were observed. The balance between LLM insights and clinical judgment, as well as continuous model refinement, is crucial.

Conclusion: LLMs like ChatGPT offer significant potential in rheumatology, providing swift and broad medical insights. However, a cautious approach is vital, ensuring rigorous training and ethical application for optimal patient care and clinical practice.

## Introduction

Artificial intelligence (AI) continues to play an increasingly important role in medical practice and technology [1]. Its advancement as a field is radically transforming the landscape of clinical care, with significant considerations and outcomes for physicians, patients, and policymakers alike [2]. With such an emerging field, it is vital to map out the different ways that it can impact informed decision-making processes. In the context of rheumatology, large language models (LLMs) such as Chat Generative Pre-Trained Transformer (ChatGPT) can personalize the retrieval of scientific information by understanding the context of a user's query and tailoring the responses to meet specific needs, such as summarizing complex scientific articles into easy-to-understand formats for informed decision-making [3]. These models can also analyze vast datasets of scientific literature to identify key insights, trends, and relationships, thereby assisting in evidence-based decision-making by providing synthesized and relevant information [4].

In the context of Sjögren's Syndrome, ChatGPT and other large language models can offer targeted insights that are particularly helpful in differentiating this disorder from other rheumatological conditions. For example, if a healthcare provider is unsure whether a patient's symptoms of dry eyes and mouth are indicative of Sjögren's Syndrome or perhaps another autoimmune condition, the model could retrieve and summarize the most current diagnostic criteria, such as the American College of Rheumatology (ACR)/European League Against Rheumatism (EULAR) classification criteria for Sjögren's Syndrome. It could highlight key serological markers like anti-SSA/Ro and anti-SSB/La antibodies, which are more commonly associated with Sjögren's than with other disorders.

The LLM could also assist in treatment planning by retrieving the latest guidelines and summarizing pharmacological options, such as the use of hydroxychloroquine for arthralgia in Sjögren's, and how that differs from its use in, say, lupus or rheumatoid arthritis. It can provide summaries of the latest studies on emerging therapies, such as B-cell targeted treatments, that may have differing efficacies in Sjögren's compared to other rheumatological diseases. For monitoring risks of non-Hodgkin lymphoma in Sjögren's Syndrome, the LLM could outline evidence-based surveillance protocols, including which imaging or laboratory tests are most indicative of disease progression or malignancy risk, as opposed to what would be relevant in other rheumatological conditions. Given these considerations, we presented four questions to ChatGPT related to Sjögren's Syndrome where the responses were evaluated for informativeness, accuracy, and overall applicability and user-friendliness by two independent reviewers. 

## Materials and methods

To explore the potential of ChatGPT in assisting healthcare professionals with insights into Sjögren's Syndrome and differentiating it from other rheumatological conditions, we developed a brief study utilizing a multi-pronged methodology. Initially, we compiled resources that consisted of peer-reviewed articles, clinical guidelines, and case studies specifically focused on Sjögren's Syndrome, as well as other rheumatological diseases. These texts were gleaned from reputable academic databases such as PubMed and ClinicalTrials.gov and were utilized to aid in analyzing the responses drawn from ChatGPT. 

The second phase involved the formulation of clinically relevant questions, by the authors, that healthcare providers commonly encounter in the diagnosis or management of Sjögren's Syndrome. Queries like "What are the immunological features of Sjögren's Syndrome?", "What are the histopathological features of Sjögren's Syndrome that make it more high risk for getting non-Hodgkin lymphoma?", "What is the appropriate follow-up management for a patient presenting Sjögren's Syndrome?", and "I have joint pain, dry eyes, dry mouth, and a persistent dry cough. What is the differential diagnosis?" were developed. These questions, four in total, were then used as prompts to engage ChatGPT, and the responses were thoroughly assessed in terms of their accuracy, relevance, and comprehensiveness, aligned with the current scientific literature. We were mindful of presenting questions in “new chats” to avoid it utilizing past conversation history to curate a specifically tailored response [5]. For example, when stating symptoms such as joint pain and dry eyes and requesting a differential diagnosis of the matter, past conversation about Sjögren's Syndrome’s immunology could influence its response as the subject matter was already being discussed [6]. 

Ensuring an unbiased assessment of the ChatGPT's utility was paramount in our study design. To achieve this, we employed multiple techniques. For one, we implemented a blinding technique, removing any tags or markers that might identify the source as an LLM [7]. This allowed for a blind evaluation by three reviewers which included clinicians with internal medicine specializations, as part of drawing from past studies making use of physician reviewers [8]. We also ensured that all evaluators declared any potential conflicts of interest prior to their assessments. The responses generated by ChatGPT were also cross-validated by repeating them in a new conversation thread to verify overall thematic consistency and accuracy. Scores from 1 (low) to 5 (high) were also independently assigned by reviewers, which were then averaged, to each article using the 15-questionnaire DISCERN tool to assess written health material [9]. To further bolster the unbiased nature of our assessment, we introduced several additional methods. Randomized control prompts about other rheumatological conditions like arthritis and osteoarthritis were interspersed with the main prompts to gauge if the model displayed any preference or bias towards Sjögren's Syndrome [10]. Temporal analysis was performed by conducting the study at various time points to examine if updates to the model influenced its response to our control queries. After finding no prominent difference in results to our control prompts between June-August 2023, we utilized ChatGPT Version 4 for our analysis which continues to regularly receive updates and technical fixes, with our focus on the end results of the Sjögren's Syndrome-related questions being based on the model present as of August 31, 2023. By rigorously adhering to these materials and methods, our study aims to offer a robust and unbiased evaluation of the capabilities of LLMs like ChatGPT in aiding the diagnosis and management of Sjögren's Syndrome in comparison to other rheumatological conditions.

## Results

**Figure 1:**
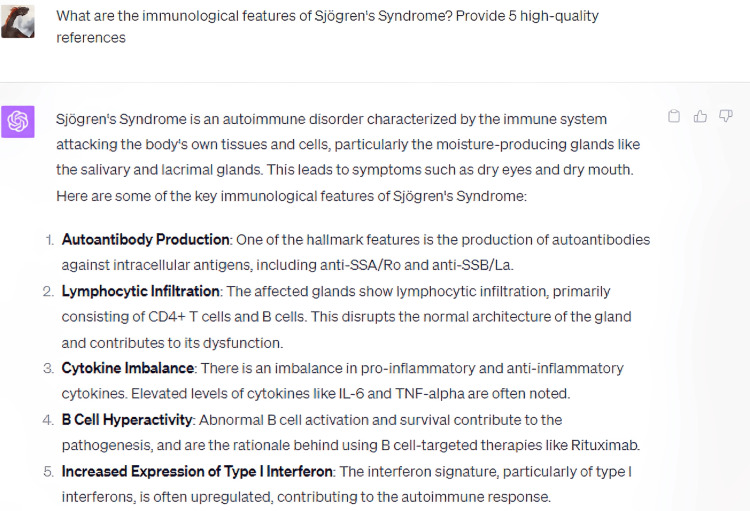
What are the immunological features of Sjögren's Syndrome?

**Figure 2:**
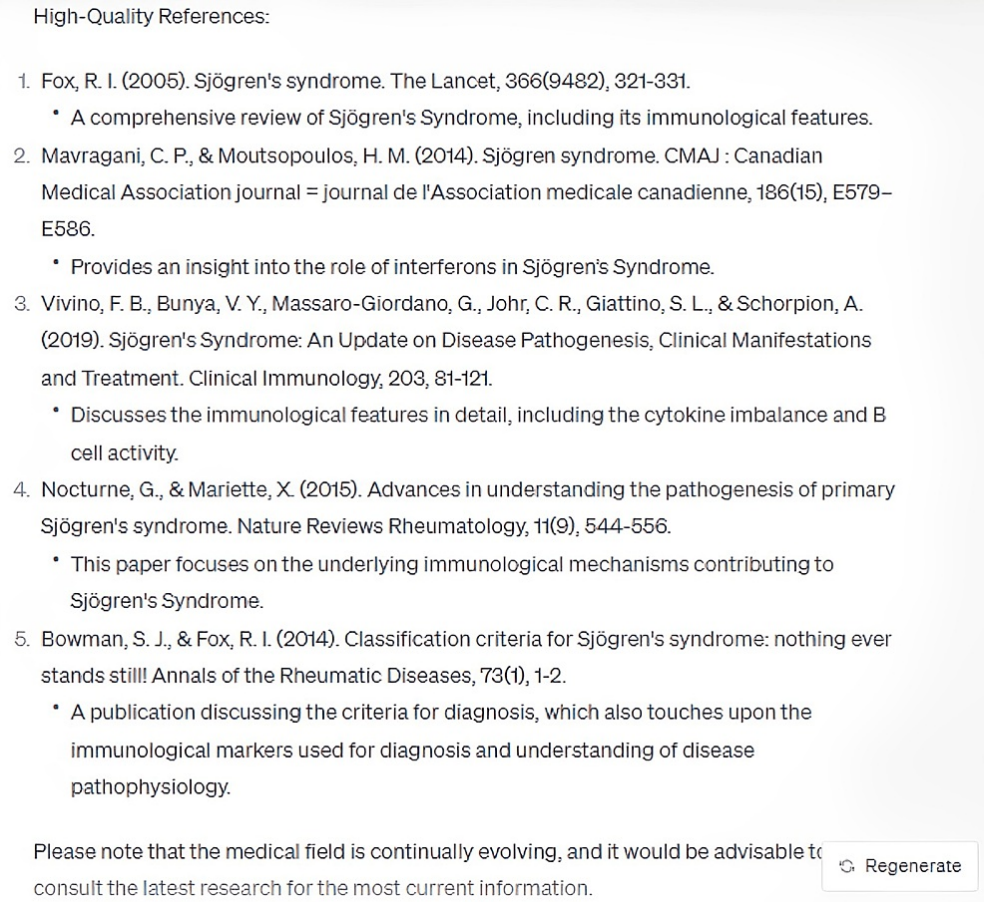
References for "What are the immunological features of Sjögren's Syndrome?"

Upon questioning ChatGPT regarding the immunopathological characteristics of Sjögren's Syndrome, it elucidated key aspects, encompassing autoantibody generation, lymphocytic infiltration, imbalances in cytokine production, B-cell hyperactivity, and an augmented expression of Type I interferon (Figure 1). Intriguingly, the model inherently proffered five salient points, independent of any preset constraints, engendering implications for potential hierarchization of these pathophysiological features in terms of significance or prevalence. The bibliographic citations generated in alignment with this query were predominantly from credible sources; however, an inconsistency was noted in the fourth reference, where the year of publication was erroneously delineated (Figure 2). Notably, ChatGPT judiciously incorporated a caveat emphasizing the dynamism inherent in medical knowledge and flagged its own knowledge cut-off at September 2021, underscoring its circumspection [11]. 

**Figure 3:**
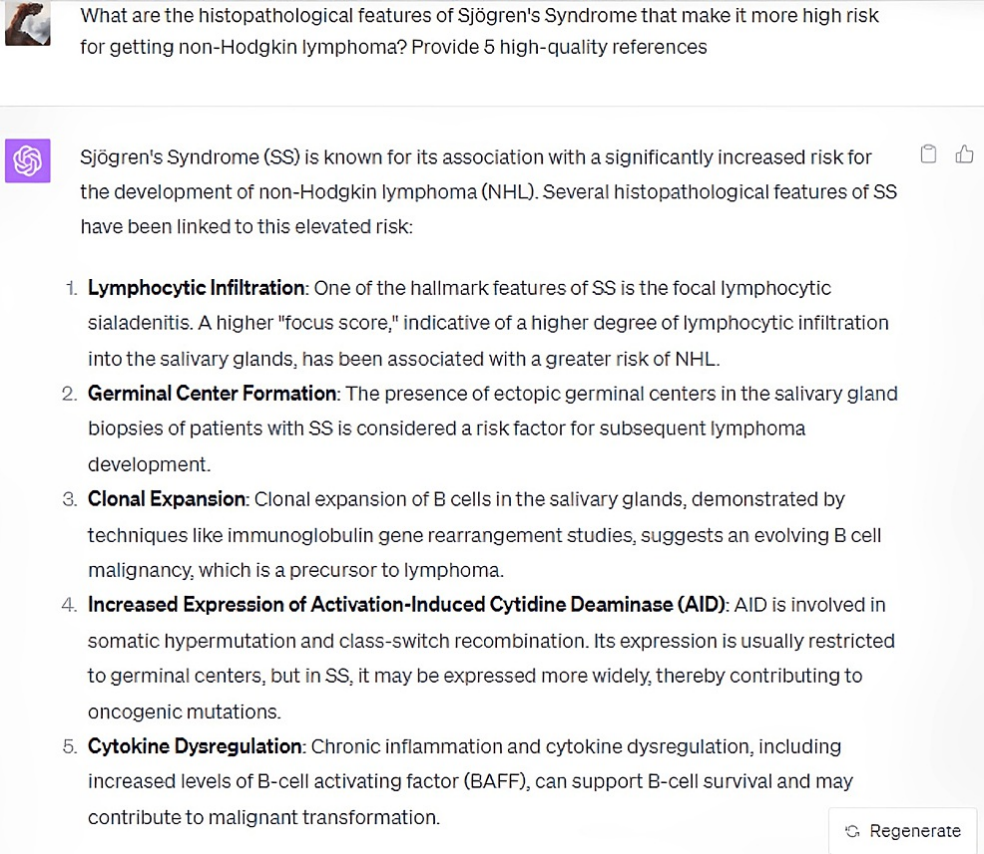
What are the histopathological features of Sjögren's Syndrome that make it more high risk for getting non-Hodgkin lymphoma?

**Figure 4:**
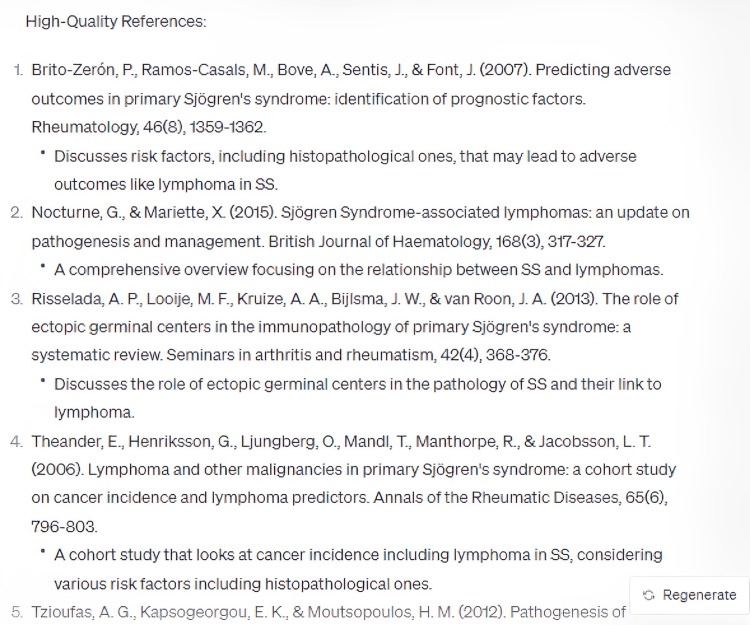
References for "What are the histopathological features of Sjögren's Syndrome that make it more high risk for getting non-Hodgkin lymphoma?" (Part 1)

**Figure 5:**
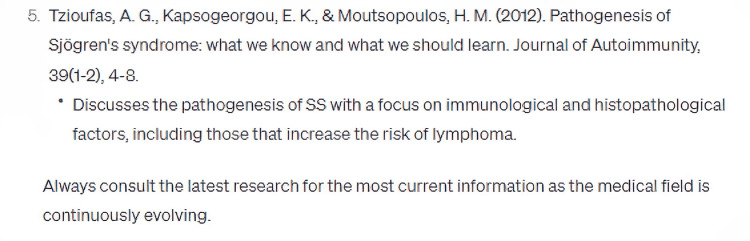
References for "What are the histopathological features of Sjögren's Syndrome that make it more high risk for getting non-Hodgkin lymphoma?" (Part 2)

**Figure 6:**
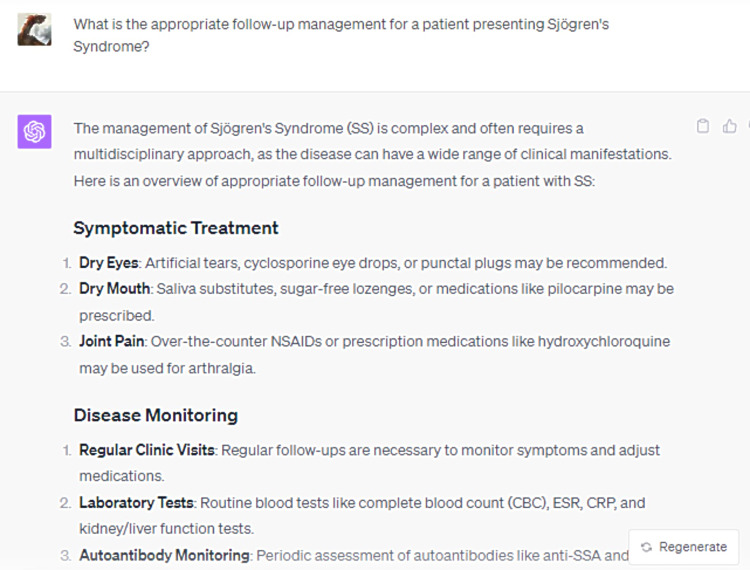
What is the appropriate follow-up management for a patient presenting Sjögren's Syndrome? (Part 1)

**Figure 7:**
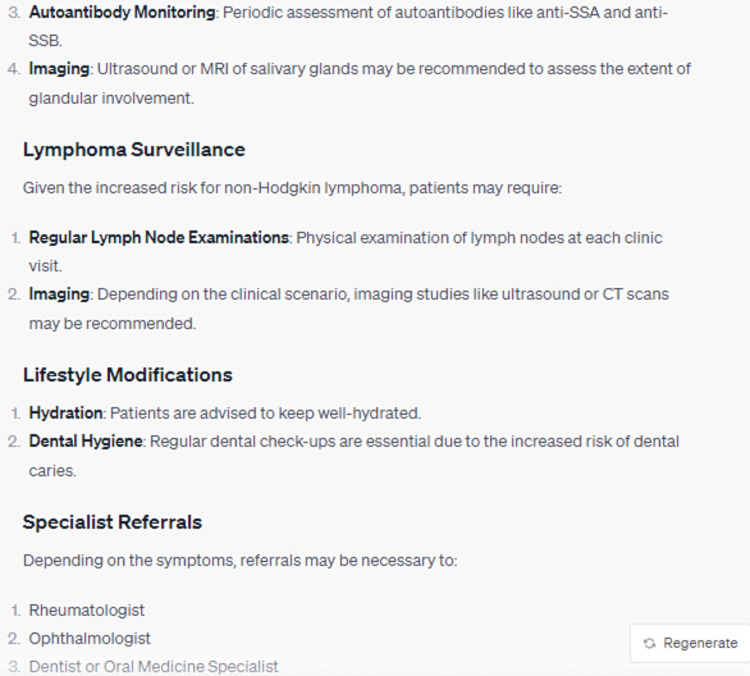
What is the appropriate follow-up management for a patient presenting Sjögren's Syndrome? (Part 2)

**Figure 8:**
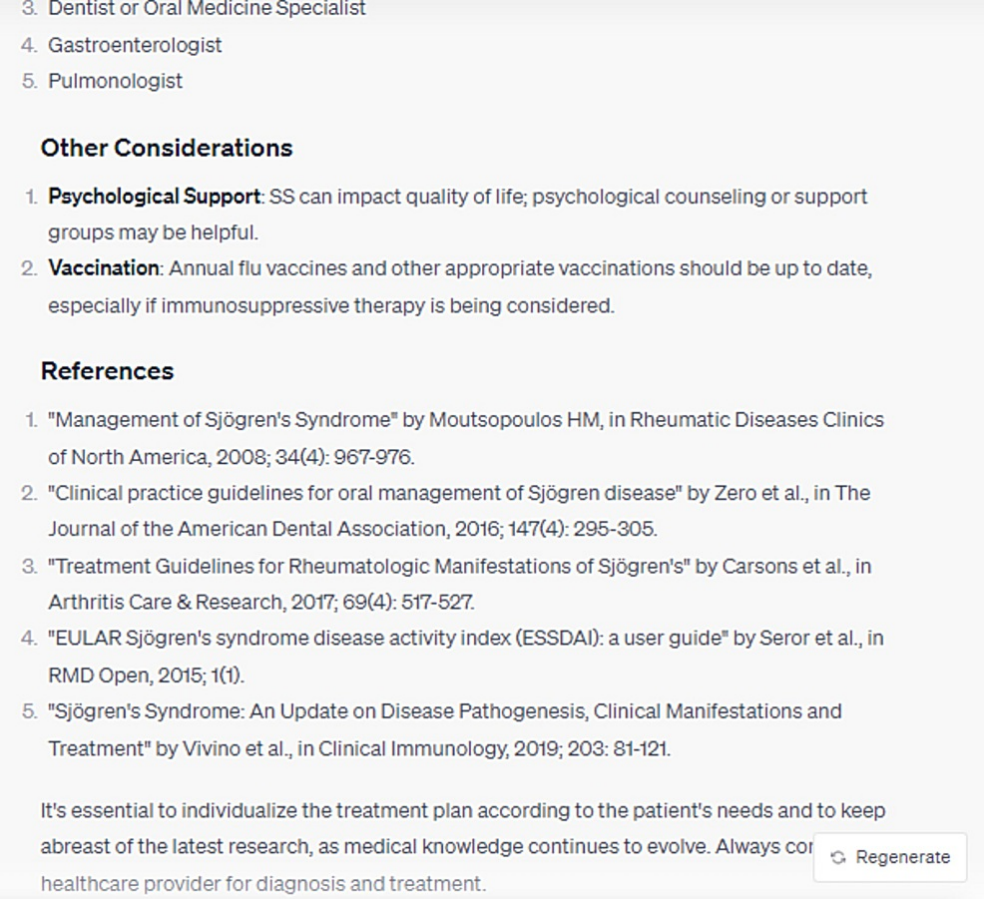
What is the appropriate follow-up management for a patient presenting Sjögren's Syndrome? (Part 3)

In its elucidation of the histopathological attributes specific to the syndrome, ChatGPT enumerated five cardinal features, whilst omitting crucial data, notably the periductal infiltration of the salivary and lacrimal glands predominantly by CD4+ helper T (Th) lymphocytes, among other cellular entities (Figure 3). Furthermore, the model did not delineate the genomic predilections contributing to the syndrome's pathogenesis. Disparities were also observed in the bibliographic citations for therapeutic management, with the foremost reference manifesting discrepancies in authorship attribution, publication chronology, title, and source journal (Figures 4-8) [12]. 

**Figure 9:**
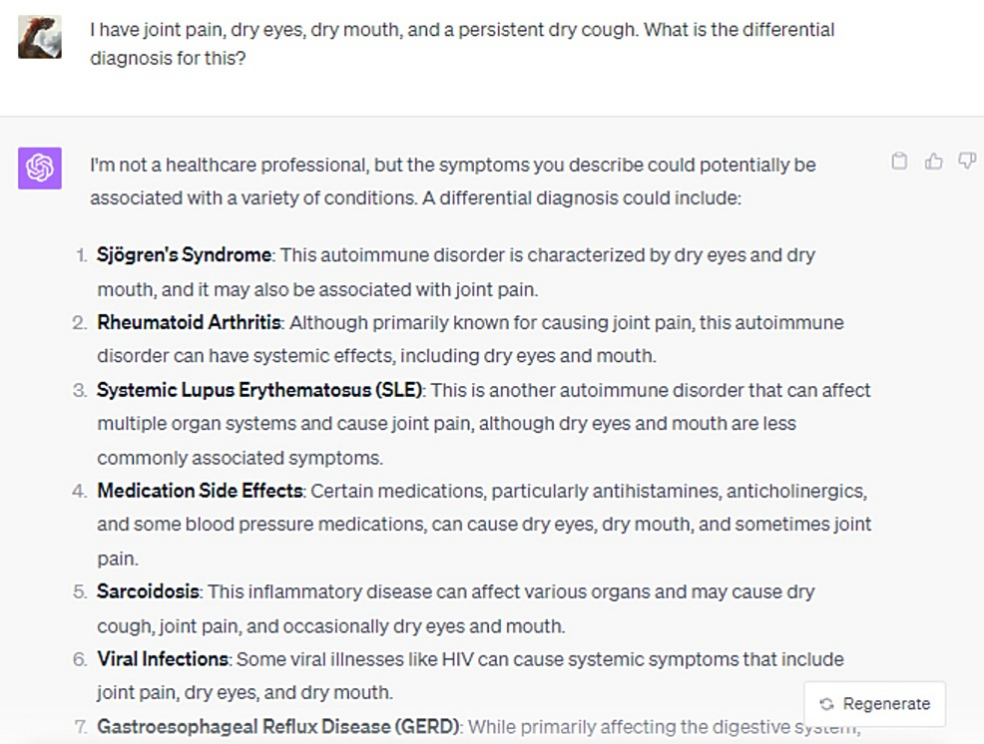
I have joint pain, dry eyes, dry mouth, and a persistent dry cough. What is the differential diagnosis for this?

**Figure 10:**
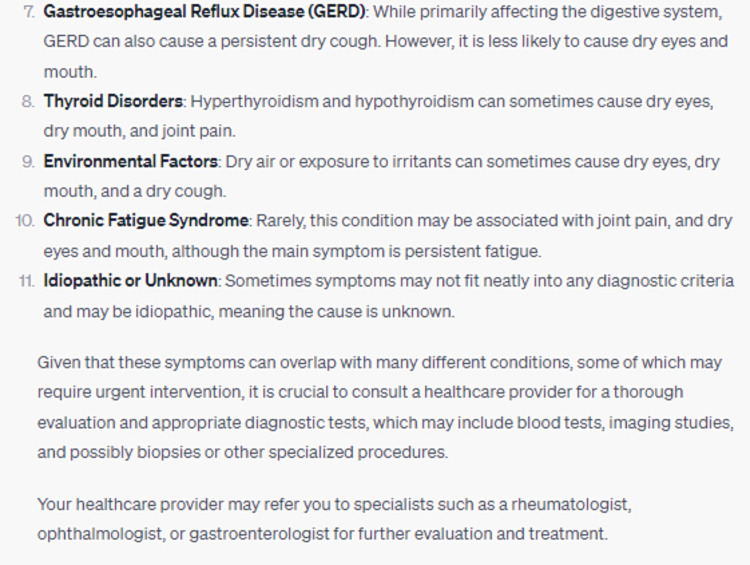
Recommendation to See Specialists

When prompted with a clinical vignette detailing xerophthalmia, arthralgia, xerostomia, and persistent dry cough, ChatGPT adeptly identified a myriad of plausible etiological considerations, with Sjögren's Syndrome being preeminent (Figure 9). Furthermore, the model sagaciously expounded on the imperativeness of a comprehensive clinical evaluation and intimated potential specialist referrals encompassing rheumatology, ophthalmology, or gastroenterology (Figure 10) [13]. DISCERN scores were also relatively high for much of the responses presented by ChatGPT (Table 1). 

**Table 1:** DISCERN Scores for ChatGPT-generated responses to questions

Question	DISCERN Score
What are the immunological features of Sjögren's Syndrome?	4.22
What are the histopathological features of Sjögren's Syndrome that make it more high risk for getting non-Hodgkin lymphoma?	3.67
What is the appropriate follow-up management for a patient presenting Sjögren's Syndrome?	3.97
I have joint pain, dry eyes, dry mouth, and a persistent dry cough. What is the differential diagnosis for this?	4.82

## Discussion

The utilization of artificial intelligence and LLMs like ChatGPT in the realm of rheumatology offers a spectrum of both promising opportunities and notable challenges. From our study, the immediacy with which ChatGPT can access and summarize vast amounts of data presents a significant advantage for both patients and healthcare providers [14]. Such access can facilitate evidence-based decision-making, particularly in complex areas like rheumatology where differential diagnosis can be intricate [15].

For patients, the use of ChatGPT can democratize information, offering insights into their symptoms and potential conditions even before a clinical consultation [16]. This can empower them with information, enabling more informed conversations with their healthcare providers [17]. For the common person, it provides a platform to understand complex medical conditions in simplified terms, bridging the knowledge gap [18]. Furthermore, for physicians and clinical providers, it can act as an efficient aid in clinical decision-making by offering quick references, diagnostic criteria, and updated treatment guidelines [19].

The limitations observed in our results, such as the tendency of ChatGPT to default to a brief explanation by listing only a select number of features, raise concerns about potential oversimplification [20]. The inherent risk is that essential clinical features may be omitted, leading to an incomplete understanding [21]. Moreover, its dependence on its last training data (in this case, up to September 2021) means it might not always provide the most up-to-date information. As seen from our results, ChatGPT did have inaccuracies in reference citations, further emphasizing the importance of cross-referencing [22].

While LLMs can simplify complex scientific information, there's an inherent responsibility to ensure that this does not inadvertently lead to misinformation [23]. As we observed, ChatGPT does acknowledge its last update, which is vital for transparency. However, there's a need for robust mechanisms to continuously update and train these models with the latest medical research. Another ethical consideration is the potential for LLMs to inadvertently influence clinical decision-making if relied upon too heavily. Physicians must balance the insights from LLMs with their clinical judgment. Additionally, it's imperative to address the potential bias in LLMs. As seen with our control queries about other rheumatological conditions, ensuring that the AI does not display any undue preference for a particular condition is crucial [24]. Overreliance on AI responses without critical evaluation can skew clinical perceptions. This is all becoming increasingly relevant as social media and telehealth technology continues to expand to refine areas of care and be a source of medical information [25,26]. 

## Conclusions

The adoption of LLMs like ChatGPT in the domain of rheumatology holds vast potential, offering swift access to a broad spectrum of medical knowledge that can enhance evidence-based clinical decision-making. Our study underscores the efficiency and utility of ChatGPT in demystifying complex medical concepts, bridging the knowledge gap for both patients and healthcare professionals. However, this potential is counterbalanced by some limitations. The oversimplification observed in some responses and occasional inaccuracies in reference citations urge a cautious approach in relying solely on LLMs without cross-referencing or incorporating clinical judgment. As artificial intelligence continues to make strides in healthcare, the continuous refinement, rigorous training, and ethical application of these tools will be paramount in ensuring their optimal use in patient care and clinical practice.
